# Long Non-Coding RNAs in the Cold-Stress Response of Horticultural Plants: Molecular Mechanisms and Potential Applications

**DOI:** 10.3390/ijms262110464

**Published:** 2025-10-28

**Authors:** Magdalena Wielogórska, Anna Rucińska, Yuliya Kloc, Maja Boczkowska

**Affiliations:** 1 Plant Breeding and Acclimatization Institute—National Research Institute, Radzików, 05-870 Błonie, Poland; m.bialoskorska@ihar.edu.pl (M.W.); a.rucinska@ihar.edu.pl (A.R.); y.kloc@ihar.edu.pl (Y.K.); 2 Botanical Garden, Center for Biological Diversity Conservation in Powsin, Polish Academy of Science, Prawdziwka 2, 02-976 Warszawa, Poland

**Keywords:** long noncoding RNA, lncRNA, cold stress, horticultural crops, gene regulation

## Abstract

Cold stress reduces horticultural crop yield and postharvest quality by disrupting membrane fluidity, redox equilibrium, and the cell wall structure. This results in chilling injury, tissue softening, and loss of color. Long noncoding RNAs (lncRNAs) have emerged as key integrators of plant cold signaling pathways. LncRNAs mediate the interaction between calcium signaling systems and transcriptional cascades while coordinating hormone signaling networks, including those involving abscisic acid, jasmonic acid, ethylene, salicylic acid, and brassinosteroids. LncRNAs influence gene regulation through chromatin-based guidance, sequestration of repressive complexes, natural antisense transcriptional interference, microRNA-centered competing endogenous RNA networks, and control of RNA splicing, stability, localization, and translation. Studies in horticultural species revealed that cold-responsive lncRNAs regulate processes essential for fruit firmness, antioxidant levels, and shelf-life, including lipid modification, reactive oxygen species balance, and cell wall or cuticle remodeling. This review aims to summarize tissue- and developmental stage-specific expression patterns and highlight experimental approaches to validate RNA function, including gene editing, transcript recovery, advanced sequencing, and analysis of protein-RNA interactions. Integrating these results will facilitate the development of precise molecular markers and nodes of regulatory networks that increase cold tolerance, and improve the quality of horticultural crops.

## 1. Introduction

Cold stress, which is caused by temperatures below 15 °C, significantly reduces the productivity, quality, and postharvest performance of horticultural crops. During storage, cold injury (CI) is a distinctive physiological problem that has an adverse effect on market value. Recent comprehensive studies integrate knowledge of CI symptoms, underlying mechanisms, and mitigation strategies for various fruits and vegetables [1]. Plants alleviate cold stress through processes of cold acclimation, which include membrane and thermosensory perception, calcium-dependent (Ca^2+^) signaling pathways, kinase cascades, and transcriptional regulation. These processes are primarily coordinated by the *Inducer of CBF Expression 1-C-repeat Binding Factors-Cold-Responsive* genes (ICE1-CBF-COR) signaling module and by reactive oxygen species (ROS) homeostasis and hormone-dependent interactions [2].

LncRNAs have emerged as pivotal regulators of plant responses to abiotic stresses, including cold stress [3]. Cold stress significantly restricts the productivity of horticultural crops. LncRNAs modulate gene expression through transcriptional regulation, chromatin remodeling, RNA stability, and interactions with small RNAs [3]. Often, lncRNAs function by interacting with transcription factors, modifying chromatin states, or acting as molecular decoys to fine-tune stress responses [4]. Understanding how lncRNAs mediate cold stress regulation in horticultural crops is expanding.

This study aims to comprehensively review and elucidate the molecular roles of lncRNAs in the cold stress response of horticultural plants. Current knowledge on the diverse regulatory functions of lncRNAs, such as transcriptional modulation, epigenetic regulation, and interaction with signaling pathways, that underpin cold tolerance is lacking. Furthermore, this study highlights recent advances in identifying and characterizing cold-responsive lncRNAs across key horticultural species. Potential applications of lncRNAs in breeding and biotechnological strategies to increase cold resilience and improve crop productivity under adverse environmental conditions are also discussed.

## 2. Plant Long Non-Coding RNAs

### 2.1. Definition

Long non-coding RNAs are transcripts that are longer than 200 nucleotides and lack significant protein-coding potential [5]. Unlike messenger RNAs, their diverse biogenesis and structural complexity make annotation and functional prediction challenging [6,7]. Once dismissed as transcriptional noise, lncRNAs are recognized as essential regulators of gene expression that modulate chromatin conformation and transcription, both locally and distally [8,9]. They also affect RNA processing, stability, translation, and nuclear architecture. Plant lncRNAs originate from intergenic, intronic, antisense, promoter-, and enhancer-proximal regions. This reflects their diverse regulatory functions [10,11]. LncRNAs often exhibit low sequence conservation across species. However, their involvement in stress responses, development, and epigenetic regulation is well documented in plants [12,13]. This opens the door for the identification of potential targets to improve plant resilience and productivity.

### 2.2. Biogenesis and Genomic Context

In plants, most lncRNAs are transcribed by RNA polymerase II and undergo processing similar to that of messenger RNAs (mRNAs), including 5′ capping, alternative splicing, and 3′ polyadenylation [14]. RNA polymerase III transcribes shorter, more stable, and more abundant lncRNAs, which undergo polyadenylation less frequently. These transcripts are involved in stress responses and immune regulation [15]. Plants possess two additional RNA polymerases IV (Pol IV) and V (Pol V), which produce lncRNAs involved in RNA-directed DNA methylation (RdDM), a process that is critical for gene silencing and genome stability [11,16]. Pol IV-derived lncRNAs are precursors of small interfering RNAs. Pol V transcripts serve as scaffolds that guide silencing complexes to chromatin, modulating its architecture and often exhibiting tissue specificity [16,17]. Thus, plant lncRNA biogenesis involves a complex interplay of multiple polymerases and posttranscriptional modifications that underpin their diverse regulatory roles in development and environmental responses.

### 2.3. Classification

**Figure 1:**
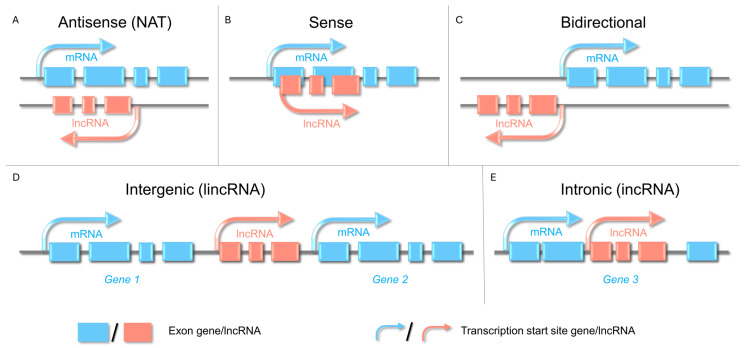
Classification of long non-coding RNAs (lncRNAs) based on the genomic context concerning protein-coding genes. (**A**) Natural antisense transcript (NAT) lncRNAs are transcribed from the strand opposite a nearby protein-coding gene. The resulting NAT overlaps exons and/or introns and can modulate sense transcription through transcriptional interference or RNA-RNA-chromatin interactions. (**B**) Sense-overlapping-lncRNAs transcribed from the same strand as a protein-coding gene, overlapping part of its exonic/intronic sequence, may therefore, interfere with the splicing, elongation, or transcript turnover rate of the mRNA. Interference may influence splicing, elongation, or RNA turnover. (**C**) Bidirectional-lncRNAs initiated from a promoter region that drives a protein-coding gene in the opposite direction (head-to-head, typically within ~1 kb of the coding TSS), enabling coordinated, strand-specific regulation. (**D**) Intergenic lncRNAs (lincRNAs) arise from an intergenic locus and do not overlap with any annotated coding genes. Regulation is conferred by its own promoter/enhancers and distal chromatin contacts. (**E**) Intronic lncRNAs are wholly contained within an intron of a host gene, do not overlap with its exons, and may be transcribed or processed independently of host transcripts.

Plant lncRNAs are classified according to their genomic context concerning protein-coding genes. This classification is crucial for understanding the origin and regulatory roles of lncRNAs. Types of lncRNAs include natural antisense transcripts (NATs), sense lncRNAs, bidirectional lncRNAs, intergenic lncRNAs (lincRNAs), and intronic lncRNAs (incRNAs) (Figure 1) [6,15]. NATs overlap with antisense genes and regulate gene expression transcriptionally and posttranscriptionally. Sense lncRNAs originate from the same strand and modulate nearby gene activity. Bidirectional lncRNAs originate near transcription start sites, but are transcribed from the opposite strand of the DNA template. LincRNAs are abundant in species such as tomato (*Solanum lycopersicum* L.) and cucumber (*Cucumis sativus* L.) and exhibit tissue specificity and diverse regulatory functions. IncRNAs originate entirely from introns and influence splicing and host gene expression [18]. These classifications reflect the dynamic and varied functions of lncRNAs in the development and stress responses of horticultural plants.

### 2.4. Mechanisms of Action

**Figure 2:**
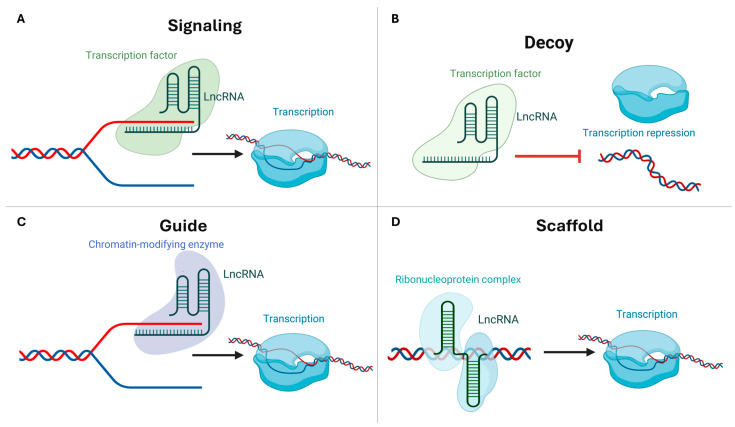
Mechanisms of action of plant long non-coding RNAs. LncRNAs regulate gene expression through four conserved modes of action: (**A**) Signaling—in response to a stimulus, such as cold, an induced long non-coding RNA (lncRNA) is transcribed. This lncRNA functions as a molecular signal that engages enhancer- or promoter-proximal regulators. This process controls the timing and location of the assembly of an active transcription complex. It also initiates the transcription of a target gene. (**B**) Decoys—lncRNAs act as decoys by sequestering a transcription factor, RNA-binding protein, or small RNA component away from its cognate DNA/RNA site. This prevents the factor from occupying the promoter and consequently modulates transcriptional output. (**C**) Guide—lncRNAs guide regulatory proteins to specific genomic locations through RNA—protein and/or RNA-DNA/RNA base-pairing interactions. This process concentrates chromatin modifiers or transcription factors at target promoters, thereby activating or repressing transcription. (**D**) Scaffolds–lncRNAs serve as modular scaffolds that carry multiple binding surfaces. These surfaces allow lncRNAs to nucleate higher-order regulatory assemblies, such as transcription factors with chromatin enzymes, at the locus. This stabilizes interactions that fine-tune transcription. This schematic diagram was edited with BioRender.

Despite rapid sequence divergence, lncRNAs in plants function through conserved modes of interaction. These RNAs act as signals, decoys, guides, or scaffolds, linking structural classes (lincRNAs, NATs, and incRNAs) to regulatory roles (Figure 2) [19,20]. Signal lncRNAs respond to environmental cues by modulating transcription via promoter remodeling and alternative RNA processing. This phenomenon has been observed in tomato and grapevine (*Vitis vinifera* L.) [21,22]. Decoy lncRNAs sequester microRNAs (miRNAs) or proteins, thereby regulating crop nutrient homeostasis and pathogen defense responses [23]. Guide lncRNAs direct chromatin modifiers to target loci, thereby modulating gene expression. COLDAIR exemplifies this process in Arabidopsis (*Arabidopsis thaliana* L.) and its homologs in horticultural species [24,25]. As demonstrated in rice (*Oryza sativa* L.) and Arabidopsis, scaffold lncRNAs organize protein complexes to facilitate chromatin remodeling [26,27]. These functional archetypes are essential for development and stress adaptation in horticultural plants.

## 3. Sensing and Signaling of Cold: Integration Nodes for lncRNAs

### 3.1. ICE1–CBF–COR Module and Coregulators

**Figure 3:**
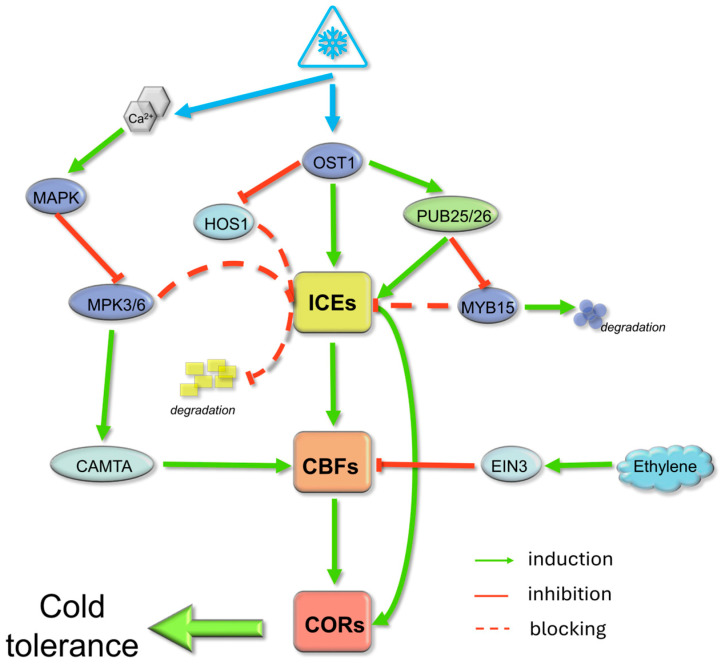
The canonical ICE1-CBF-COR regulatory cascade is the basis of plant responses to cold acclimation. Upon exposure to cold, ICE (Inducer of CBF Expression) transcription factors are activated, triggering the rapid induction of C-repeat Binding Factors (CBFs). These CBFs then upregulate *Cold Responsive* (*COR*) genes, enhancing cold tolerance. OST1 kinase phosphorylates ICEs, stabilizing them and promoting *CBF* transcription. HOS1-mediated ubiquitination facilitates ICE degradation, modulating response intensity. PUB25/26-mediated ubiquitination stabilizes ICEs and inhibits MYB15, which is a negative regulator of *CBF*s. The MAP kinases MPK3/6 further fine-tune ICE stability by promoting destabilization through phosphorylation-dependent mechanisms, thereby creating negative feedback. Calcium influx activates CAMTA transcription factors, which can also induce *CBF*s. Ethylene and its signaling component EIN3 act as negative regulators by inhibiting *CBF*s. Induction, inhibition, and signal blocking are represented by green, red, and dashed lines, respectively, integrating various signaling components into a multilayered cold response regulatory network.

The ICE1-CBF-COR cascade forms the basis of transcriptional regulation in response to cold. ICE1 initiates the rapid induction of dehydration-responsive element-binding (DREB)/CBF transcription factors, which subsequently activate *COR* genes while undergoing complex multilayer regulation (Figure 3) [28]. After cold exposure, Snf1-related kinase 2.6 (SnRK2.6)/OST1 (open stomata 1) becomes activated and phosphorylates ICE1. This increases ICE1 stability and simultaneously inhibits HOS1-dependent degradation. Thus, *CBF* expression increases during the early stages of acclimation [29]. In turn, the MAP kinases MPK3 and MPK6 phosphorylate ICE1, promoting its destabilization and creating a delayed negative feedback loop that attenuates the response [30]. Temporal regulation is further enhanced by differential ubiquitination. Early attachment of K63-linked ubiquitin chains by PUB25/26 stabilizes ICE1 and inhibits MYB15. Later, K48-linked ubiquitination attenuates the signal [31]. Calcium ion (Ca^2+^) influx activates calmodulin-binding transcription activator (CAMTA) transcription factors that bind to *CBF* promoters, integrating Ca^2+^-mediated signals with light and diurnally regulating *CBF* expression [28]. Similar regulatory networks are evident in horticultural systems. For example, multiomic analyses in banana (*Musa* spp.) revealed a mitogen-activated protein kinase Kinase 1 (MEKK)1-MAPK-ICE1-COR signaling axis coupled to lipid remodeling that contributes to cold tolerance [32].

### 3.2. Hormonal and Metabolic Interactions

Phytohormones integrate the ICE1 node into physiological networks throughout the plant. Abscisic acid (ABA) generally enhances frost tolerance by interacting with the OST1-ICE1 pathway. Ethylene signaling, which is mediated by ethylene-insensitive 3 (EIN3), often opposes *CBF* transcription. On the other hand, jasmonic acid (JA) exerts a context-dependent effect by modulating the balance of growth and defense. Brassinosteroids (BRs) can promote cold tolerance. The overexpression of *SIBRI1* in tomato elevates ICE1/CBF transcripts and stimulates ROS detoxification pathways [33]. From a metabolic point of view, cold stress disrupts membrane fluidity and redox homeostasis. Tolerant genotypes typically exhibit increased unsaturation of glycerolipids and increased antioxidant capacity. In bananas, the accumulation of specific lipid forms coincides with the activity of the MAPK-ICE1-COR47 axis [32]. These hormonal and metabolic processes converge on the ICE1-CBF pathway and its parallel pathways to fine-tune the amplitude and duration of the cold response.

### 3.3. Hypothesized and Documented lncRNA Points of Action

LncRNAs are involved in various layers of cold signaling integration, including chromatin targeting and decoy functions involving Polycomb complexes. For example, the antisense lncRNA SVALKA from Arabidopsis restricts *CBF1* expression through transcriptional interference and potentially modulates Polycomb-mediated chromatin states at the *CBF* locus [34]. Transcriptional interference is also mediated by NATs, as demonstrated in chrysanthemum (*Chrysanthemum morifolium* Ramat.). NATs increase *DgTCP1* expression, increasing cold tolerance and confirming this mode of regulation in ornamental plants [35]. Competing endogenous RNAs (ceRNAs) mimic miRNA target receptors. Specifically, lncRNAs act as sponges for miRNAs, alleviating the repression of stress-responsive transcription factors. Although cold-specific modules are still being elucidated, this molecular mechanism has been established in tomato and suggested for cucurbits subjected to cold stress, highlighting its importance for horticultural adaptation to cold [36,37]. The scaffolding role in the RdDM and small-interfering RNA (siRNA) pathways involves Pol V-linked lncRNAs that recruit Argonaute (AGO)-siRNA complexes and the DNA methyltransferases Domains Rearranged Methyltransferase 1/2 (DRM1/2) to deposit cytosine methylation in regulatory regions. This enables epigenetic tuning of inducible promoters [7,38,39]. Additionally, posttranscriptional regulation affects the splicing and stability of cold-responsive transcripts [3]. Together, these pathways establish lncRNAs as versatile modulators capable of recalibrating the ICE1-CBF cascade and its hormonal and redox interfaces in vegetable, fruit, and ornamental plant species.

## 4. LncRNA Mechanisms of Action in Cold Stress

### 4.1. Epigenetic and Transcription Regulation

Acclimation to cold requires the rapid elimination of Polycomb repression of specific genes. In Arabidopsis, under short-term chilling, many cold-induced loci translocate histone H3 lysine 27 trimethylation (H3K27me3) under controlled conditions and accumulate histone H3 lysine 4 trimethylation (H3K4me3) when exposed to cold. This is consistent with the stabilized chromatin architecture and the recruitment or translocation of lncRNA-mediated chromatin modifiers [40]. In plants, lincRNAs can engage Polycomb repressive complex 2 (PRC2)/LHP1 and DNA methylation readers for chromatin reprogramming. The Arabidopsis lncRNA APOLO forms R-loops on target promoters, binds LHP1 and VIM1 (a UHRF1 homolog), and coordinates H3K27me3 with DNA methylation to regulate genes in response to the environment. This establishes a general RNA scaffold that can then guide the response of cold-induced modules [41].

The biology of plant enhancers introduces a vital nuance: unlike mammals, unstable bidirectional enhancer RNAs (eRNAs) are rare in plants. The Interspecies Transcription Atlas revealed that distal bidirectional unstable transcription, a characteristic of vertebrate eRNAs, is uncommon. Instead, many distal plant regulatory elements produce stable RNAs, often lncRNAs, and exhibit more vigorous enhancer activity in Self-Transcribing Active Regulatory Region sequencing (STARR-seq) than unstable transcripts do [42]. Therefore, “enhancer-like” lncRNA mechanisms in plants should be inferred based on functional enhancer assays (and 3D contact data), not just bidirectional instability. Finally, NATs can fine-tune transcription through polymerase interference. In chrysanthemum. For example, the NAT controlling *DgTCP1* modulates cold tolerance, illustrating the role of NATs in horticulture [35].

### 4.2. Crosstalk with Small RNAs

LncRNAs bind to miRNA pathways as endogenous target imitators or ceRNAs. In cucumber, CsLncRNA94 forms a cold-responsive module with miR156f and *Squamosa Promoter Binding Protein-like* (*SPL*) mRNAs; transient overexpression alters miR156f/*SPL* expression patterns and reduces cold tolerance, which is consistent with a miRNA titration mechanism affecting developmental/stress centers [43]. Robust inference of ceRNAs in plants requires plant-trained target predictors and degradome support for the miRNA-mRNA cleavage site [44,45]. Horticultural syntheses catalog cold-coupled lncRNAs, which are predicted to sponge miRNAs that target desaturases or antioxidant enzymes, linking membrane fluidity and ROS detoxification to the CBF regulon—hypotheses that can now be tested via Parallel Analysis of RNA Ends (PARE)-supported target maps and genetic epistasis [4].

### 4.3. Posttranscriptional Control

Cold stress alters alternative splicing (AS), mRNA stability, and translation. Plant lncRNAs can bind splicing regulators to reprogram alternative splicing (AS) programs. Recent reviews of plants integrate such lncRNA-AS interactions with stress phenotypes, suggesting a pathway for tuning signaling and metabolic isoforms during periods of reduced temperature [7]. At the stability/translation interface, plants undergo cotranslational mRNA decay, which links ribosome transit to mRNA degradation and the stress response. LncRNAs incorporated into ribonucleoproteins (RNPs) or associated with polysomes can disrupt the selective stability or subcellular route of cold-responsive mRNAs [46]. Since some annotated lncRNAs contain translated small open reading frames (sORFs), the assignment of a cold phenotype must exclude micropeptide effects by integrating Ribo-seq (triplet periodicity), ORF-specific mutagenesis (start-codon disruption), and proteomics. Conversely, if a micropeptide is produced, gain or loss at the peptide level should phenocopy the effect [47]. Emerging publications also include lncRNAs among the RNAs that organize biomolecular condensates (Liquid-Liquid Phase Separation (LLPS)), a likely mechanism for rapid acclimation to cold temperatures. However, direct evidence in plants remains limited and should include LLPS markers and lncRNA disruption [7].

## 5. Case Studies: Horticultural Plants

### 5.1. Grapevine (Vitis vinifera L.)

**Figure 4:**
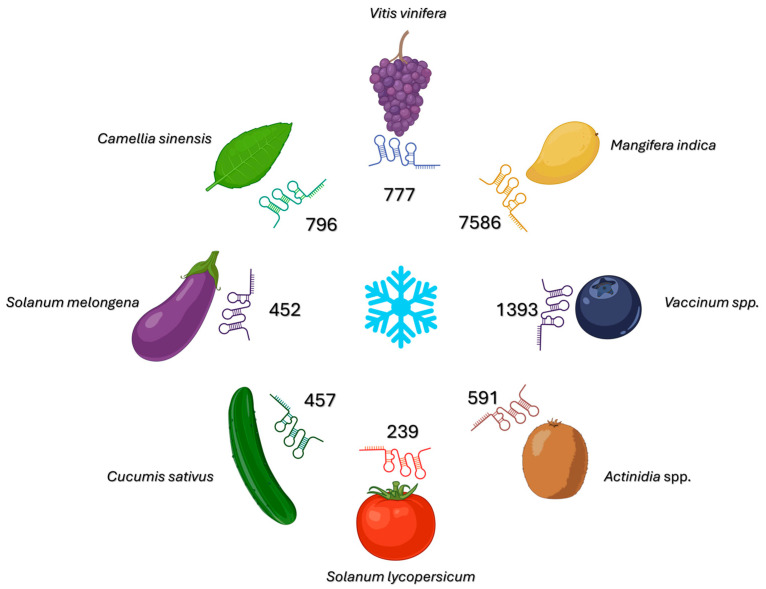
Cold-responsive long non-coding RNAs (lncRNAs) across horticultural species. This radial summary illustrates the number of lncRNAs identified as cold-responsive (i.e., differentially expressed under chilling/freezing conditions versus control conditions) in *Vitis vinifera, Mangifera indica, Vaccinium* spp., *Actinidia* spp., *Solanum lycopersicum*, *Cucumis sativus*, *Solanum melongena* and *Camellia sinensis*. The central snowflake denotes the low-temperature stimulus. The colored “RNA” symbols are schematic markers of lncRNAs and are illustrative only. The numeric labels provide the quantitative information. The counts compile the totals reported in the cited RNA-seq studies for each species and may reflect study-specific pipelines, tissues, developmental stages, and stress regimes. Therefore, cross-species comparisons should be interpreted cautiously. This schematic diagram was edited with BioRender.

The sequencing of RNA specific to grapevine leaves exposed to cold temperatures revealed hundreds of cold-responsive (CR)-lncRNAs (Figure 4). Many of these RNAs, such as *C-repeat Binding Factor 4* (*CBF4), Late Embryogenesis Abundant 14* (*LEA14)*, and *WRKY41*, are *cis*-linked to proximal cold pathway genes. Coexpression analyses combined with RNAplex-based pairing have confirmed functional relationships, both *cis-* and *trans*-, between these transcripts [48]. The evidence supporting their regulatory role is primarily computational and suggests the *cis*-activation of the *CBF* and *LEA* gene modules. However, the experimental validation of their enhancer-like functions remains inconclusive [48]. The predicted mRNA targets include those encoding CBF/DREB transcription factors, LEA proteins, calcium ATPases, and UDP-glucosyltransferases (UGTs). Several CR-lncRNAs have been found to overlap with genomic regions associated with chloroplast and mitochondrial genes. This suggests that communication between organelles and the cell nucleus occurs during cold acclimation [48,49]. There are no reports of phenotypes resulting from molecular manipulation strategies for grapevine CR-lncRNAs; however, the modules with which they bind are enriched for cold-tolerant genotypes in interspecific crosses and correspond to physiological processes, such as cell membrane stabilization and osmoprotection, observed during acclimation [48,49]. Potential applications include marker-assisted selection via CR-lncRNA-CBF/LEA haplotypes, editing the promoters of *cis*-acting CR-lncRNAs to modulate ICE-CBF signaling with minimal pleiotropic effects, and predicting cultivar-specific responses to low-temperature storage on the basis of network analyses of horticultural crops [49].

### 5.2. Kiwifruit (Actinidia spp.)

Storage of kiwifruit at low temperatures after harvest activates lncRNA programs involved in starch and sucrose metabolism and cell wall remodeling. These processes are associated with maintaining fruit firmness and mitigating frost damage. These findings stem from integrated multiomics studies of fruits and vegetables [37]. Network analyses in kiwifruit revealed that enhancer-like lncRNAs and ceRNA motifs may participate in the regulation of carbohydrate metabolism by influencing the expression of genes such as expansins, pectin methylesterase/lyase (PME/PG) enzymes, and sugar transporters. The full-length transcriptomes of kiwifruit under cold conditions revealed that lncRNAs are coexpressed with cold-responsive transcription factors [50]. Full-length transcriptome sequencing under cold conditions further showed coexpression of lncRNAs with cold-responsive transcription factors. Analysis of molecular interaction networks indicates that lncRNAs, miRNAs, and mRNAs can be interconnected within modules that regulate the expression of these target genes. Transcription factors from the CBF and DOF/MYB families appear to be coregulated in cold datasets [37]. These correlations link these lncRNA modules to phenotypic traits such as firmness preservation and reduced chilling damage. However, functional perturbations at the gene level have yet to be described. Potential applications include designing storage regimes based on lncRNA expression profiles associated with cell wall turnover and pretreatment with MeJa or ABA before harvesting to activate lncRNA–ceRNA networks and stabilize cell wall integrity and reactive oxygen species homeostasis [37].

### 5.3. Citrus (Citrus spp.)

*Citrus* species undergo robust transcriptomic reprogramming under cold stress. Although direct functional characterization of CR-lncRNAs remains limited, lncRNA atlases have been generated for lemon (*Citrus* × *limon*) and other species [51,52,53]. Cold-induced activation of anthocyanin biosynthesis programs through transcription factors, such as Ruby1, which is regulated by CBF-coupled factors, is a key pathway that lncRNAs can modulate through interactions with ceRNA or chromatin-mediated mechanisms. This is analogous to data from other fruit crops [54]. The documented cold-responsive networks in *Citrus* include those involving transcription factors and ROS-scavenging enzymes. LncRNAs have been functionally mapped for secondary metabolism and disease resistance, suggesting a transferable framework for cold-response research [51]. Existing lncRNA catalogs can be translated into a reanalysis of cold response datasets by comparing NATs and coexpression modules with metabolomic and pigment data. NATs that resemble those in the CBF or ICE1 loci and those that affect anthocyanin accumulation and antioxidant protection could be prioritized as regulatory hubs that link abiotic tolerance to fruit quality traits. Such integrative analyses can reveal lncRNA–miRNA–mRNA interaction axes that regulate cell wall remodeling, osmolite metabolism, or the fine-tuning of ROS, using degradome and small RNA datasets. These data analyses can enable marker-assisted selection or CRISPR-based modulation of key NAT loci associated with cold tolerance and high nutritional quality. The targeted manipulation of these lncRNAs or their associated promoters is an easily applicable method for identifying the epiballistic and transcriptional variants that underlie adaptive cold tolerance. Breeding high-quality, cold-tolerant citrus varieties that combine stress resistance with optimal pigmentation and antioxidants could be made easier by translating the signatures of long non-coding RNA-based regulatory systems into a breeding framework.

### 5.4. Mango (Mangifera indica)

A computational analysis of various mango cultivars revealed over 31,000 candidate lncRNAs, of which more than 7500 exhibited significant expression changes at chilling temperatures (5–12 °C) (Figure 4) [55]. These cold-responsive lncRNAs target genes involved in critical pathways, such as metabolic regulation, stress response, and development. These pathways include *Alcohol Dehydrogenase 1* (*ADH1*) and *3-ketoacyl-CoA thiolase 2 (KAT2)*, which play a role in fruit ripening and abscisic acid signaling. Network analysis revealed their interactions with WRKY transcription factors that are central to cold tolerance. Functional enrichment analysis links these lncRNAs to photosynthesis, sugar metabolism, flavonoid biosynthesis, and membrane processes. Some lncRNAs exhibit conserved sequences in related species, indicating that their regulatory roles are conserved [55]. Mango lncRNAs form complex networks that regulate physiological and metabolic adaptations to cold. This makes them promising targets for breeding cold-tolerant mango varieties.

### 5.5. Blueberries (Vaccinium spp.)

Transcriptomic and proteomic analyses of blueberries under cold stress provide fundamental insights into the regulatory circuits underlying acclimation. Although lncRNA and their regulatory layers have not been fully characterized in existing cold-stress datasets, their abundance during fruit development in related systems suggests the value of retrospective exploration of lncRNAs within existing RNA-seq data [56,57]. Translational efforts should focus on reannotation of available cold stress libraries via lncRNA identification and ceRNA network methods, followed by validation of strong lncRNA candidates that correlate with key phenotypes, such as cell membrane integrity and antioxidant defense, associated with reduced fruit pitting and softening.

### 5.6. Tomato (Solanum lycopersicum)

Strand-specific RNA sequencing of chilled tomatoes revealed more than 1400 lncRNAs, 239 of which were differentially expressed during chilling injury (Figure 4). Co-expression analyses revealed modules associated with cell membrane structure, ROS homeostasis, cell wall-modifying enzymes, heat and cold shock proteins, and salicylic acid (SA)/ABA metabolism [58]. Integrated analyses revealed a network of competing endogenous RNAs at the lncRNA-miRNA-mRNA level that respond to cold injury. Degradome data confirmed that ceRNAs and target mimicry occur via miRNA targeting and anti-correlated expression profiles. These modules focus on lipid remodeling, ROS detoxification, and defense hormone regulation [58]. The predicted interactions of miRNA families target AGO1, antioxidant genes such as copper/zinc superoxide dismutase (CSD), and cell wall enzymes. Stress-related transcription factors such as DREB/CBF and NAC domain transcription factors are also localized in these networks [59,60]. Network rearrangement is consistent with physiological tolerance indicators, such as reduced electrolyte leakage, malondialdehyde content, and pitting, which are observed in tolerant lines or after elicitor treatment. Although the gene-specific functional validation of cold-responsive lncRNAs (CR-lncRNAs) in tomato remains limited, several established molecular approaches can be employed. These include overexpression and RNA interference (RNAi) assays, virus-induced gene silencing (VIGS), and antisense or hairpin constructs for the transient or stable suppression of lncRNA activity. These routine functional genomics tools provide direct phenotypic evidence and can be complemented by allele-specific CRISPR interference (CRISPRi) or promoter editing expression fine-tuning is needed. Their practical application extends beyond genome editing. It also includes transgenic complementation, promoter–reporter assays, and overexpression in tolerant genetic backgrounds. Together, these offer a versatile framework for identifying and manipulating lncRNA nodes. These nodes predict cold tolerance and post-harvest quality stability [61,62].

### 5.7. Eggplant (Solanum melongena)

LncRNAs play a key role in regulating the cold stress response in eggplant, and genotype-specific expression patterns have been linked to variations in tolerance. Transcriptomic analyses of the cold-tolerant “CGN22911” and cold-sensitive “Chengdumoqie” varieties revealed 452 differentially expressed lncRNAs under low-temperature conditions (Figure 4) [63]. These lncRNAs modulate target genes involved in metabolic and cellular processes essential for cold adaptation, such as acyl-CoA dehydrogenase and pseudouridine synthase activities. The enriched pathways included oxidative phosphorylation, peroxisome function, endoplasmic reticulum protein processing, and ubiquitin-dependent proteolysis, which emphasize cellular homeostasis during cold stress. The marked enrichment of pathways between genotypes reveals the mechanisms underlying differential tolerance. Many lncRNAs regulate genes involved in ROS metabolism, cell membrane stability, and protein quality control through *cis*- and *trans*-interactions, thereby coordinating complex networks of stress responses [63]. Notably, the two varieties presented different enrichment patterns, suggesting the existence of specific lncRNA regulatory circuits that may underlie the variation in cold tolerance. The dataset provides prioritized lncRNA candidates for functional validation through *cis/trans* target analyses and ceRNA interaction studies, offering promising entry points for regulatory genetic improvement and breeding of cold-tolerant eggplant cultivars.

### 5.8. Cucumber (Cucumis sativus)

The responses of cucumber to coding regulators such as *basic pentacysteine 2* (*CsBPC2)*, *HRS1 homolog 2 (CsHHO2)*, and *galactinol synthase 1* (*CsGolS1)* are well understood [64,65,66]. Recently, cucumber lncRNA repertoires exhibiting temperature-sensitive expression profiles, including the downregulation of specific lncRNA expression under cold stress, have been described [43,67]. While a direct functional role for cold-sensitive lncRNAs has yet to be confirmed experimentally, promoter analyses and transcriptome dynamics have identified candidate lncRNAs associated with abscisic acid-, jasmonic acid-, and ROS-related subnetworks involved in cold tolerance. These predicted ceRNA associations link lncRNAs to genes involved in hormone signaling and cell membrane integrity, with several cold-induced transcription factors (e.g., BPC, HHO, and MYB families) thought to interact [43,64,65,66,67]. Combining the potential lncRNAs identified via the omics method with confirmed cold tolerance effectors offers a multilayered improvement strategy to reduce fruit cold damage in the production and supply chain phases.

### 5.9. Chrysanthemum (Chrysanthemum morifolium)

The natural antisense transcript DglncTCP1, which is transcribed antisensely to the *teosinte branched1/cycloidea/proliferating 1* (*DgTCP1)* gene, has been characterized in chrysanthemum. This lncRNA acts as a scaffold that recruits trithorax-like H3K4 methyltransferase (DgATX), which antagonizes the Polycomb 2 repressive complex. This increases the number of H3K4me3 marks at the *DgTCP1* locus, thereby increasing *DgTCP1* expression [35]. RNA immunoprecipitation and chromatin isolation confirmed this model. The transcription factor DgTCP1 directly activates *DgPOD*, which facilitates the removal of ROS. The overexpression of *DglncTCP1* or *DgTCP1* increases freezing tolerance. In contrast, CRISPR-mediated knockout of *DgTCP1* decreases tolerance. These results prove that RNA-mediated chromatin modulation improves cold acclimation in ornamental species [35]. This NAT-mediated chromatin recruitment strategy is promising for enhancing frost hardiness in ornamental plants while minimizing its impact on growth.

### 5.10. Tea (Camellia sinensis)

In tea, the gradually expanding catalog of non-coding RNAs, including lncRNAs, has been linked to secondary metabolism and responses to temperature stress [68,69]. Transcriptomic analyses revealed that many lncRNAs that are differentially expressed under abiotic stress conditions share signaling components with those under cold stress, such as the regulation of ROS and calcium signaling pathways. For example, the coexpression of specific lncRNAs with calcium-transporting ATPases suggests their involvement in Ca^2+^ signaling cascades, which are crucial for acclimation to cold [68]. Functional predictions and network analyses suggest that tea lncRNAs modulate gene expression by acting as ceRNAs for microRNAs and interacting with transcription factors to influence hormone signaling pathways relevant to cold tolerance. While direct experimental verification of the function of lncRNAs in tea-induced cold stress is limited, the conserved role of identified lncRNAs in regulating stress-related transcription factors, ROS homeostasis, and metabolic pathways suggests their importance in the cold response [68]. These studies highlight the potential of using tea lncRNA profiles for molecular breeding and improving cold stress resistance. Translational approaches will likely benefit from integrated multimaps and functional assays that elucidate the mechanisms of lncRNA regulation under cold stress.

## 6. Prospects for Application and Challenges for lncRNA-Based Cold Stress Applications in Horticultural Crops

In light of recent studies, lncRNAs can be key regulators of adaptation to cold stress, coordinating the transcriptional and physiological processes underlying crop resilience. An increasing number of studies from horticultural species suggest that lncRNAs play a role in post-harvest cold tolerance and storage quality by influencing ROS removal pathways and hormonal interactions [70]. The broad regulatory network highlights the function of lncRNAs as dynamic molecular switches, coordinating the multifactorial responses essential for cold acclimation and stress memory. Characterizing them has a dual benefit: it deepens our understanding of cold tolerance and allows for the definition of specific molecular targets for breeding climate-resilient horticultural crops [71].

### 6.1. Application of Molecular Tools

In horticultural crops, cold-responsive lncRNAs can be organized into RT-qPCR panels and embedded in genetic designs that treat lncRNA abundance as a quantitative trait [72]. Expression quantitative trait loci (eQTLs) and related association frameworks, which are already routine for ripening and texture, can be extended to lncRNAs to identify loci whose expression covaries with chilling injury indices, firmness decay, and antioxidant balance, especially when long reads and allele-aware mapping resolve isoforms [73,74]. Recent resources and studies have explicitly incorporated noncoding transcripts into eQTL/Transcriptome-Wide Association Studies (TWAS) analyses, underscoring the feasibility of this strategy. For candidates with supportive evidence, *cis*-regulatory edits (promoter or enhancer motifs using base/prime editors) allow dosage tuning without altering the protein-coding sequence. Moreover, CRISPR activation (CRISPRa) systems provide potent transcriptional upregulation from native loci. The CRISPR-Act3.0 platform achieves robust, multiplex activation across Arabidopsis, rice, and tomato and can be coupled to PAM-relaxed nucleases to broaden the target space [75]. Similarly, CRISPRi repression using dCas9-based repressors has matured to reversible gene-circuit toolkits and improved repressor designs, enabling systematic down-tuning and orthogonal controls in planta [76]. Together, these platforms support causal testing of lncRNA-centered hypotheses in relevant tissues and developmental windows. Multiple biostimulant and edible-coating regimes alleviate cold-storage deterioration: MeJA reduces chilling injury across species according to a quantitative meta-analysis; combinations of MeJA or SA with 1-Methylcyclopropene (1-MCP) improve firmness and limit weight loss; and chitosan-based coatings (including nanoformulations) help maintain quality in tomato, mandarin (*Citrus reticulata* Blanco) and other fruits [77,78,79,80,81]. While direct cause–effect links between these treatments and specific lncRNA modules remain limited, lncRNA expression panels are well-suited for monitoring readouts to evaluate intervention efficacy across cultivars and storage regimens.

### 6.2. Critical Biological Limitations

Robust inference requires RNA-centered designs that leave local DNA context intact. In plants, CRISPRa can uptune transcription from native promoters (e.g., CRISPR-Act3.0 enables multiplex activation across Arabidopsis, rice, and tomato), whereas CRISPRi using improved dCas9 repressors provides stable downtuning; together, they permit bidirectional tests of function without altering the underlying locus. Trans-rescue from an ectopic site should restore the candidate RNA to confirm specificity because specific transcripts annotated as lncRNAs harbor short open reading frames (sORFs) producing bioactive micropeptides, Ribo-seq and deep proteomics, along with start-codon/frameshift-mutant rescues that preserve RNA structure, are necessary to exclude peptide-driven phenotypes [75,82,83]. Plant lncRNA loci frequently express multiple isoforms with distinct ends, structures, and interactomes. Long-read RNA-seq (PacBio Iso-Seq or ONT DRS) is indispensable in defining the operative isoform(s) before perturbation. Functional tests should target isoform-unique features, splice junctions, polyadenylation signal (PAS)/cleavage sites, or isoform-specific exons, and pair loss-of-function with isoform-matched rescue. Given potential redundancy among paralogous noncoding transcripts or nearby cis-regulatory elements, tiling approaches and multiplex perturbations may be needed to reveal phenotypes of modest effect size [84,85]. Many validated lncRNA effects are organ- and stage-restricted. The signals detected in the leaf during cold acclimation often fail to reproduce in fruit during post-harvest storage. Therefore, experimental designs should consider different time points in ontogeny, tissue types, and genotypes. For hybrids or grafted pairs, allele-aware mapping is necessary to assign signals unambiguously. Under stress conditions, the movement of mRNA from the rootstock to the scion was demonstrated in studies of Cucurbits, implicating mobile transcripts in cold-related responses [86,87]. However, a comprehensive meta-analysis indicated that the global extent of long-distance mRNA communication has likely been overestimated. It emphasizes mapping biases, inadequate genotype controls, and the limited use of reciprocal grafts [88]. Consequently, claims of mobility and exceptionally functional long-distance lncRNA movement should satisfy stringent criteria. These include SNP-aware pipelines, reciprocal grafts, vascular time series, strict k-mer/UMI-based chimera filters, and donor tissue perturbation yielding concordant receiver tissue changes.

### 6.3. Technological and Translational Barriers

The number of elite cultivars and woody perennials remains difficult to transform. Two classes of regeneration boosters have changed the landscape: morphogenic factors *WUSCHEL* (*WUS*) and *BABY BOOM* (*BBM*), which promote somatic embryogenesis and markedly increase transformation efficiency in cereals; and Growth-Regulating Factor (GRF)–GRF-Interacting Factor (GIF) chimeras, which accelerate shoot formation and broaden the scope of genotypes in cereals and citrus. Despite these gains, responses remain genotype-dependent and can entail pleiotropic growth effects that must be mitigated by inducible or transient expression [89,90,91]. Plant-tailored CRISPRa/CRISPRi systems now enable the reversible modulation of native loci, for example, CRISPR-Act3.0 for the multiplexed activation of genes, and new dCas9-based repressors that are effective in Arabidopsis and cucumber [75]. These systems support the testing of hypotheses without altering the underlying DNA sequence. Near-PAMless SpRY increases the range of targets in rice and other plants, but can also increase the risk of off-target effects, necessitating high-fidelity variants and careful guide design [82,92,93]. Prime editing continues to improve through the tuning of DNA repair and design rules; however, efficiencies vary across species [94]. Virus-based delivery can bypass recalcitrant tissue culture. It can increase HDR. This is achieved using TRV/PVX/FoMV vectors and rhabdovirus/geminivirus platforms. However, cargo limitations, host range, and inheritance remain key constraints [95,96]. Progress is slowed by inconsistent naming and by overreliance on primary-sequence similarity in cross-species analyses. Plant lncRNAs show low sequence conservation yet often retain positional (syntenic) conservation; comparative inferences should therefore prioritize genomic position, promoter context, and conserved RNA structures over raw nucleotide identity [97]. Adopting coordinate-based identifiers (including strand and isoform tags), aligned with curated resources (PLncDB, GreeNC), would improve portability across studies [98,99]. In parallel, functional annotation should incorporate RNA-binding protein (RBP) interaction maps using plant-adapted ribonomics (e.g., HyperTRIBE, RIP/eCLIP-like workflows) and dsRNA-binding protein profiling, which provide orthogonal evidence that a candidate engages specific protein partners under stress [100,101]. Addressing these barriers requires pairing context-controlled regeneration (WUS/BBM, GRF–GIF) with precision modulation and delivery (CRISPRa/CRISPRi, SpRY, prime editing, viral/replicon systems), while enforcing naming standards that reflect how plant lncRNAs evolve and function. Such alignment will enable rigorous, comparable studies of lncRNAs in cold-stress biology across species and genotypes of horticultural plants [90].

### 6.4. Outlook and Knowledge Gaps

Three limitations currently constrain robust inference for lncRNAs in the cold-stress response of horticultural plants. First, causality in plants is limited. RNA-centered perturbations that leave the locus intact, i.e., CRISPR activation/repression at native promoters, complemented by antisense or dCas13 approaches, should be coupled to *trans*-rescue and to the systematic exclusion of peptide effects via Ribo-seq/proteomics and start-codon/frameshift controls. The Arabidopsis SVALKA/SVALNA case illustrates how multi-layer evidence is needed even for canonical cold-induced loci [75,102]. Second, isoform complexity and redundancy are underaddressed. Plant lncRNA loci frequently express multiple isoforms with distinct ends and interaction profiles. Long-read RNA-seq improves the recovery of major isoforms and should precede perturbation; functional tests should target isoform-unique junctions or 3′ ends and pair loss-of-function with isoform-matched rescue. Community-level benchmarks confirm the superior isoform resolution of long reads and highlight the need for consistent reporting standards in plants [85]. Third, portability across organs and the reality of long-distance RNA movement remain uncertain. The signals detected in the leaf during cold acclimation rarely extrapolate to the fruit pericarp during storage; designs should integrate time-resolved sampling and tissue stratification, with allele-aware mapping in hybrids or grafts. For graft systems, convincing long-distance movement of lncRNAs in horticultural species has not been established [88]. Standardization is a cross-cutting need. Because primary-sequence conservation is low, cross-species inference should weight genomic position and synteny, promoter context, and RBP interaction data over raw sequence identity. Adopting coordinate-based, strand- and isoform-aware identifiers and linking them to curated repositories would improve portability and reduce study ambiguity. Differential expression catalogs, qRT-PCR panels, co-expression modules, initial VIGS/RNAi assays, CRISPRa/CRISPRi in model and select crops, and enhancer assessment with STARR-seq are routinely used today. However, isoform-resolved, RNA-centered causality with rescue; multienvironment validation in fruit tissues; rigorous mobility pipelines; harmonized nomenclature/metadata; and broader transformation/delivery solutions in recalcitrant horticultural genotypes still require further development.

## 7. Conclusions and Perspectives

LncRNAs have emerged as integrative regulators within plant cold signaling networks. These genes link calcium/CAMTA inputs and the ICE1-CBF-COR transcriptional cascade with hormonal pathways, chromatin remodeling, sRNA-mediated interactions, and posttranscriptional regulation. In horticultural species, cold-responsive lncRNAs are consistently associated with modules that control membrane lipid remodeling, ROS homeostasis, and cell wall and cuticle dynamics. These processes collectively influence key quality attributes, such as firmness, coloration, antioxidant capacity, and shelf-life [7,37]. Comprehensive analyses of cold signaling pathways highlight how they intersect and can be fine-tuned during acclimation. This defines regulatory nodes through which lncRNAs modulate the intensity and duration of responses [103,104].

From an applied perspective, two avenues are promising. First, profiling lncRNA expression across tolerant and sensitive cultivars provides quantitative information that complements morphological and eQTL-based trait analyses, offering predictive insights into cold adaptability [37]. Second, transcriptional engineering strategies enable fine-tuned modulation of gene dosage without altering protein-coding capacity. CRISPR activation systems, for example, have been applied to induce *CBF4* transcription in grapevine, resulting in reduced electrolyte leakage and increased cold tolerance [105]. These results underscore not only technical feasibility but also the regulatory flexibility of activating key endogenous cold-response hubs. By coupling CRISPRa or CRISPR interference with the manipulation of non-coding loci, such as promoters harboring lncRNA–miRNA interaction sites, it becomes possible to modify stress response kinetics rather than structural gene output. This targeted dosage control provides an experimental framework for testing hypotheses on network resilience and adaptive transcriptional plasticity.

Future progress relies on integrating isoform-resolved transcriptome profiling via long-read RNA sequencing with targeted, RNA-centric perturbation assays and standardized data reporting. This will advance long non-coding RNAs from descriptive indicators toward actionable components in the breeding of cold-resilient, quality-focused horticultural crops [2,7,103,104].

## Data Availability

No new data were created or analyzed in this study. Data sharing is not applicable to this article.
